# Association between salt taste sensitivity threshold and blood pressure in healthy individuals: a cross-sectional study

**DOI:** 10.1590/1516-3180.2019.0166.R1.02102019

**Published:** 2020-02-27

**Authors:** Jéssica Martinelli, Simara Rufatto Conde, Aline Ramos de Araújo, Aline Marcadenti

**Affiliations:** I Registered Nutritionist, School of Nutrition, Biological and Health Sciences Center, Universidade do Vale do Taquari (UNIVATES), Lajeado (RS) Brazil.; II MSc. Registered Nutritionist, School of Nutrition, Biological and Health Sciences Center, Universidade do Vale do Taquari (UNIVATES), Lajeado (RS) Brazil.; III MSc. Registered Nutritionist, Postgraduate Program on Nutrition Sciences, Universidade Federal de Ciências da Saúde de Porto Alegre (UFCSPA), Porto Alegre (RS), Brazil.; IV PhD. Professor, Postgraduate Program on Health Sciences (Cardiology), Instituto de Cardiologia, Fundação Universitária de Cardiologia (IC/FUC), Porto Alegre (RS); Professor, Postgraduate Program on Nutrition Sciences, Universidade Federal de Ciências da Saúde de Porto Alegre (UFCSPA), Porto Alegre (RS); and Researcher, HCor Research Institute, Hospital do Coração (IP-HCor), São Paulo (SP), Brazil.

**Keywords:** Taste threshold, Sodium, Arterial pressure, Hypertension, Body weight, Waist circumference, Sodium intake, Potassium intake, Salt taste sensitivity

## Abstract

**BACKGROUND:**

Hypertension is an important public health problem. Overweight and high salt intake are risk factors for its development.

**OBJECTIVE:**

To evaluate the association between salt taste sensitivity threshold (STST) and blood pressure (BP) in healthy adults.

**DESIGN AND SETTING:**

Cross-sectional study conducted in a private institution.

**METHODS:**

104 healthy adults (aged 18-59 years) were evaluated. Sociodemographic, clinical and dietary data were collected. Nutritional status and BP were assessed using body mass index (BMI), waist circumference (WC), systolic blood pressure (SBP) and diastolic blood pressure (DBP). STST was assessed using graded saline solutions with sodium chloride concentrations ranging from 0.228 to 58.44 g/l. Identification of salty taste in solutions ≥ 3.652 g/l was used as the cutoff point for high STST.

**RESULTS:**

Participants with high STST presented higher daily average intakes for energy (2017.4 ± 641.5 versus 1650.5 ± 357.7 kcal/day; P = 0.01) and sodium (3070.2 ± 1195.1 versus 2435.2 ± 963.6 mg/day; P = 0.01) and higher BMI (P = 0.008) and WC (P = 0.002). After adjustment for age, sex, sodium and potassium intake, WC and family history of hypertension, the averages for SBP and DBP in subjects with high STST were higher than in those with normal STST (SBP: 138.2 ± 1.7 versus 119.7 ± 0.9 mmHg; P < 0.001; DBP: 81.2 ± 1.9 versus 75.1 ± 1.0 mmHg; P = 0.008).

**CONCLUSION:**

High STST was associated with elevated blood pressure in healthy adults, regardless of other risk factors for hypertension.

## INTRODUCTION

Hypertension is a multifactorial clinical condition in which the prevalence differs according to the ethnicity and age group of the population evaluated.^1^ The genesis of hypertension involves a number of well-described factors, such as inadequate diet (high amounts of dietary sodium and low potassium intake) and excess adiposity.^2^^,^^3^ However, the organic response to sodium overload is a mechanism that deserves attention.^4^

Epidemiological studies have suggested that susceptible individuals present elevated blood pressure (BP) through high sodium intake.^5^^,^^6^ Salt sensitivity is influenced by genetic factors, and not all the population seems to benefit from severe sodium restriction.^7^ Meta-analyses and longitudinal studies have suggested that, in comparison with adequate salt intake (4 to 5 g/day), very low sodium intake is associated with increased mortality and cardiovascular events.^8^^,^^9^ In addition to age and ethnicity, the status of BP levels (normal or elevated/hypertension) seems to be a determinant of the organic response to saline intake.^10^

The salt taste sensitivity threshold (STST) consists of the individual’s ability to recognize the taste of sodium. Taste sensitivity decreases according to age and it has been observed that this decrease begins when individuals are around 20 years old.^11^ A high STST suggests that the individual is more likely to have excessive salt intake; on the contrary, a normal STST defines that the individual is more likely to have low salt intake. It has been speculated that hypertensive individuals have greater STST than normotensive individuals, which would contribute towards higher sodium intake and, consequently, elevation of BP.^12^

Although the evidence suggests that very low salt intake may contribute negatively to cardiovascular outcomes, reducing sodium intake is still an important public health recommendation as a preventive measure against the incidence of hypertension and as therapy for those who are already affected by this disease.^13^^,^^14^ However, the relationship between STST and BP levels has not been completely elucidated in different populations, since decreased sodium consumption seems to exert better effects on BP in hypertensive black and Asian ethnic populations.^10^

## OBJECTIVE

The aim of this study was to evaluate the association between salt taste sensitivity, systolic blood pressure (SBP) and diastolic blood pressure (DBP) in clinical practice among healthy adults without hypertension, of predominantly Caucasian ethnicity.

## METHODS

A cross-sectional study was carried out between June 2015 and April 2016 among adult students and workers at a university located in southern Brazil, which is a region characterized by European colonization. The sample, which was selected for convenience, consisted of 104 adults of both sexes, aged between 18 and 59 years. Individuals with previous medical diagnoses of hypertension, diabetes mellitus or chronic kidney disease, along with pregnant women, were excluded. The study was approved by the local research ethics committee (CAAE 34989914.9.0000.5310) on September 15, 2014, and the subjects consented to their participation in the study by signing a consent statement.

The participants completed a questionnaire that the researchers had structured, which asked for sociodemographic information (age, sex, self-reported ethnicity, schooling level and income level) and clinical information, including known previous diseases and family history of hypertension (father, mother and grandparents).

To verify the frequency of consumption of food rich in sodium, a specific food frequency questionnaire (FFQ) was used. The instrument asked about 15 foods, and the participants reported the frequency of consumption of each of them, on linear scales marked out as follows: 1 - I never eat this; 2 - I eat this less than once a month; 3 - I eat this one to three times a month; 4 - I eat this once a week; 5 - I eat this two to four times a week; 6 - I eat this once a day; or 7 - I eat this twice or more per day. This questionnaire was developed among low-income Brazilian hypertensive patients and its reliability and validity were tested in the same population.^15^

In addition to the FFQ, a 24-hour food recall (R24h) was used. In this method, the participants reported all types and quantities of food consumed over the 24 hours preceding the collection of information, using domestic measurements. The information obtained from the FFQ and R24h was processed using the DietWin® 2011 nutritional software. The total daily energy intake (TEI, in kcal), macronutrients (carbohydrates, proteins and lipids, as percentages of TEI) and dietary sodium and potassium micronutrients (in mg) were evaluated.

The participants’ body mass was measured on a mechanical scale (Welmy®, model 110 CH; Santa Bárbara d’Oeste, SP, Brazil), with a maximum capacity of 150 kg, located on a flat surface and away from walls. This assessment was performed in accordance with the guidelines of the Brazilian Ministry of Health (BMH).^16^ These state that the individual needs to be positioned at the center of the calibrated equipment, barefoot, with the minimum of clothes and accessories possible and in an erect posture with feet together and arms extended alongside the body. The subject should remain standing in this position until the weight reading has been made.

Height was measured using an anthropometric ruler with a scale of 2 m, attached to the balance. For this measurement, the individual was kept standing, without shoes, in an erect posture with knees and heels together and arms extended alongside the body, and with the head positioned in accordance with the Frankfurt plan. With the subject in this position, the anthropometer piece at a right angle to the ruler was placed on the top of this individual’s head. The measurement was then made with the subject’s back, buttocks and head resting against the vertical plane of the anthropometer, in accordance with the BMH recommendations. The participants’ nutritional status was determined according to their body mass index (BMI, in kg/m^2^).

Waist circumference (WC) was used to evaluate abdominal obesity. It was measured using an inelastic anthropometric measuring tape (Cescorf®; Porto Alegre, Brazil) of 0.1 mm precision and 2 m length. This was placed horizontally at the midpoint between the lower edge of the last rib and the superior iliac crest of each participant. The subjects were placed in a standing position with their abdomen and arms relaxed. The WC criteria adopted for men and women were those established by the Brazilian Society of Cardiology (BSC).^17^

The BP (SBP and DBP) was measured with the participants at rest, in a relaxed sitting position, with uncrossed legs, feet flat on the floor and back resting on the chair. The measurements were made with subject’s arm at heart height, free from clothes, with the palm of the hand facing up and elbow slightly flexed, using a properly calibrated device (Omron®, model HEM-710 INT; Kyoto, Japan). Two consecutive readings were made, at a two-minute interval, and the second measurement was used for classification. BP was recorded as a continuous variable, and values greater than or ​​equal to 140/90 mmHg (SBP/DBP) were considered elevated.^17^

To evaluate the STST, nine solutions of sodium chloride (NaCl) were used: 1) 4 mmol/l = 0.228 g/l; 2) 8 mmol/l = 0.456 g/l; 3) 15 mmol/l = 0.913 g/l; 4) 30 mmol/l = 1.826 g/l; 5) 60 mmol/l = 3.652 g/l; 6) 120 mmol/l = 7.305 g/l; 7) 250 mmol/l = 14.610 g/l; 8) 500 mmol/l = 29.220 g/l; and 9) 1,000 mmol/l = 58.440 g/l. The solutions were manipulated in a laboratory of dietetic techniques, using potable water and analytical balance for solute measurements. They were placed in closed bottles and were kept in a dry environment without light, at room temperature.

The subjects were warned not to smoke, eat or brush their teeth over a period of at least two hours preceding the test. Four drops of the test solution were applied to the tip of the individual’s tongue. After 10 seconds without breathing or closing his or her mouth, the subject wrote on a card what the taste felt like. The solutions were offered in increasing concentrations until the individual correctly identified the taste that was felt. After making the correct identification, solutions of decreasing concentrations were then tested, until an error of identification occurred. The concentration immediately higher than this was considered to be the NaCl recognition threshold (STST). Individuals with normal STST were those who identified the salty taste in solutions 1 to 4 (≤ 1.826 g/l of NaCl [30 mmol/l]), while individuals with high STST were those who identified salty taste in solutions 5 to 9 (≥ 3.652 g/l of NaCl [60 mmol/l]). To avoid possible adaptations of the taste sensors, the tests were not done with successive concentrations, but randomly, until the identification. Between the tests, the subjects washed out their mouth with potable water.^12^^,^^18^

Data were entered into an Excel® worksheet and statistical analyses were performed in the Statistical Package for the Social Sciences (SPSS®), version 17.0 for Windows. Continuous variables were described as averages and standard deviations, and categorical variables as absolute numbers and frequencies. For comparisons among means, Student’s t test was used; and among proportions, Fisher’s exact test was used. Correlations were assessed using Pearson’s correlation test. Analysis of covariance (ANCOVA) was used to investigate the association between STST and mean blood pressure values, with adjustments according to age, sex, WC, family history of hypertension, sodium intake and potassium intake. The significance level was taken to be 5%.

## RESULTS

The average age of the participants was 28.6 ± 7.4 years; 80.8% were female and 98% were self-reportedly of white ethnicity. Among the participants, 51% had completed higher education, and 69.2% reported having a per capita income of up to three Brazilian minimum wages per month. Regarding the family history, 31.7% had no family members with any history of hypertension, 49% had up to three relatives with hypertension and 19.2% had four or more relatives with hypertension.

The average BMI of the participants was 24.1 ± 3.7 kg/m²; among them, 2.9% were considered underweight, 62.5% had normal weight, 28.9% were overweight and 5.8% were obese. The average WC was 76.9 ± 10.2 cm, and 78% of the participants presented normal values. The means for SBP and DBP were, respectively, 123.8 ± 12.3 mmHg and 76.4 ± 9.5 mmHg, and 89.4% of the participants presented normal BP.

Regarding STST, 77.9% presented normal STST and 22.1% presented high STST. Table 1 shows the characteristics of the sample according to the STST classification. There was no significant difference regarding sociodemographic variables or family history of hypertension in relation to the STST groups. However, participants classified as having higher sensitivity thresholds presented higher mean SBP and DBP, compared with the individuals who were classified as having lower thresholds (P < 0.001).

**Table 1.** Characteristics of the sample according to the salt taste sensitivity threshold classification (n = 104)

	Normal STST (n = 81)	High STST (n = 23)	P-value
Age (years)	28.1 ± 7.0	30.7 ± 8.5	0.13*
Sex (%)			0.14‡
Women	84.0	69.6	
Men	16.0	30.4	
Skin color (%)			0.40‡
White	98.8	95.7	
Schooling (%)			0.64‡
Incomplete higher education	50.6	43.5	
Completed higher education	49.4	56.5	
Family history of hypertension (%)	65.4	78.3	0.31‡
Systolic blood pressure (mmHg)	118.9 ± 8.2	141.1 ± 8.1	< 0.001*
Diastolic blood pressure (mmHg)	74.4 ± 8.6	83.61 ± 9.0	< 0.001*

*Student’s t test; ^‡^Fisher’s exact test. STST = salt taste sensitivity threshold.

There was no difference in SBP (P = 0.19) or DBP (P = 0.09) in relation to the presence or absence of a family history of hypertension. Comparing the sexes, it was observed that the mean BMI among men (26.2 ± 3.1 kg/m²) was significantly higher (P = 0.003) than the BMI among women (23.6 ± 3.7 kg/m²). The same relationship was observed regarding WC, for which the mean was significantly higher (P < 0.001) among men (88.1 ± 9.8 cm) than among women (74.3 ± 8.4 cm). Men also presented significantly higher (P = 0.001) SBP (131.9 ± 12.2 mmHg) than women (121.9 ± 11.6 mmHg), whereas there was no significant difference between the sexes regarding DBP (men: 77.9 mmHg; women: 76.1 mmHg; P = 0.43). There was no correlation between age and SBP (P = 0.22). However, the correlation between age and DBP was positive and significant (r = 0.25; P = 0.01). Similarly, the correlations between SBP and BMI (r = 0.39; P < 0.001), SBP and WC (r = 0.47; P < 0.001), DBP and BMI (r = 0.30; P = 0.002) and DBP and WC (r = 0.30; P = 0.002) were positive and significant.

Table 2 presents a comparison of dietary and anthropometric data according to the STST classification. The TEI identified among participants with high STST was significantly higher (P = 0.01) than among subjects with normal STST. No significant difference was observed in relation to the consumption of carbohydrates, proteins and lipids, according to STST levels. The sodium intake of the subjects with high STST was significantly higher (P = 0.01) than that of the subjects with normal STST. There was no significant difference in relation to potassium consumption. The BMI of the individuals with high STST was significantly higher than that of the individuals with normal STST (P = 0.008), and this was also found in relation to WC (P = 0.002).

**Table 2.** Dietetic and anthropometric data of the sample according to the salt taste sensitivity threshold classification (n = 104)

	Normal STST (n = 81)	High STST (n = 23)	P-value*
Total daily energy intake (kcal/day)	1650.5 ± 357.7	2017.4 ± 641.5	0.01
Carbohydrate (% from TEI)	50.3 ± 8.1	50.6 ± 7.9	0.86
Protein (% from TEI)	18.7 ± 4.8	17.3 ± 4.5	0.19
Total fat (% from TEI)	30.8 ± 7.6	31.9 ± 7.0	0.53
Dietary sodium (mg/day)	2435.2 ± 963.6	3070.2 ± 1195.1	0.01
Dietary potassium (mg/day)	2116.8 ± 725.5	2219.2 ± 774.5	0.56
Body mass index (kg/m^2^)	23.4 ± 2.9	26.6 ± 5.0	0.008
Waist circumference (cm)	74.8 ± 8.1	84.6 ± 13.3	0.002

*Student’s t test. TEI = total daily energy intake; STST = salt taste sensitivity threshold.

**Figure 1.**
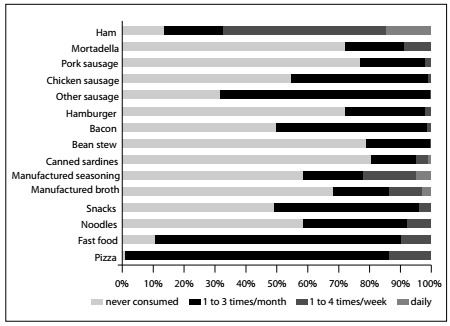
Frequency of consumption of foods with high sodium content in the entire sample (%; n = 104).

In evaluating the consumption of foods with high sodium content in the entire sample, ham, sausage, pizza and snacks had the highest frequencies, as shown in Figure 1. However, no differences were observed in the averages for SBP and DBP in relation to the frequency of consumption of foods with high sodium content. There was also no difference regarding the classification of salt taste sensitivity (normal or high) in relation to the frequency of consumption of these foods (data not shown).

Table 3 shows the SBP and DBP values according to salt taste sensitivity and adjusted for possible confounding factors. The BP values remained higher among individuals with high STST than among those classified as having normal sensitivity, after adjustment for age and sex. The same was observed after adjustments for sodium and potassium intake, WC and family history of hypertension, thus suggesting that an association existed between salt taste sensitivity and BP levels, regardless of other factors relating to BP elevation.

**Table 3.** Adjusted means for arterial blood pressure according to the salt taste sensitivity threshold classification (mean ± standard error; n = 104)

	Systolic blood pressure			Diastolic blood pressure		
	Normal STST (n = 81)	High STST (n = 23)	P-value*	Normal STST (n = 81)	High STST (n = 23)	P-value*
Model 1	119.1 ± 0.9	140.4 ± 1.7	< 0.001	74.55 ± 1.0	83.05 ± 1.8	< 0.001
Model 2	119.2 ± 0.9	140.1 ± 1.7	< 0.001	74.79 ± 1.0	82.52 ± 1.9	< 0.001
Model 3	119.7 ± 0.9	138.5 ± 1.7	< 0.001	74.99 ± 1.0	81.56 ± 1.9	0.004
Model 4	119.8 ± 0.9	138.2 ± 1.7	< 0.001	75.12 ± 1.0	81.15 ± 1.9	0.008

*ANCOVA = analysis of covariance. STST = salt taste sensitivity threshold.

Model 1: Means adjusted for age and sex; Model 2: Means adjusted for model 1 + dietary sodium and potassium intake; Model 3: Means adjusted for model 2 + waist circumference; Model 4: Means adjusted for model 3 + family history of hypertension.

## DISCUSSION

In our study, we observed an association between STST and BP levels among young adults of primarily Caucasian origin, regardless of other factors relating to BP elevation, such as age, sex, sodium and potassium intake, WC and family history of hypertension. We also, as expected, identified positive correlations between BP and obesity indicators. Moreover, we observed higher energy and sodium intake among participants with high STST, as well as higher BMI and higher WC. We did not identify any difference in relation to the presence of a family history of hypertension, according to STST status. In addition, there was no difference in SBP and DBP values ​​in relation to the presence or absence of a family history of hypertension.

In Indian adolescents, higher STST and higher BP levels were observed in individuals with a family history of hypertension.^19^ It is known that there is a genetic predisposition associated with salt taste perception and that certain populations are genetically more susceptible to development of hypertension.^20^^,^^21^ Thus, these conditions may be genetically connected, but this association needs to be better explored.^22^

The relationship between excess adiposity and sodium intake has been explored. In our study, we identified that both the BMI and the WC of participants with high STST were significantly higher than in those with normal STST, as also was dietary sodium intake. Some authors have identified a positive association between high sodium intake (detected through 24-hour urinary excretion) and changes in body composition in Caucasian populations, with increased body fat and decreased lean mass, regardless of energy intake.^23^ Other authors have shown that, for each additional 1 g of ingested salt, the chance of developing obesity is about 26%, regardless of energy intake and ethnicity.^24^ Obese individuals have lower STST than do non-obese individuals.^25^ However, salt intake appears to be higher among overweight children and adults than among those with normal body mass.^24^

High sodium intake can lead to increased food and energy intake, and it may replace the satiety effect promoted by dietary fats.^26^ In our study, we identified higher energy intake among participants with high STST. The mechanisms relating to this association may include elevation of plasma ghrelin concentrations in diets with high sodium content (this hormone acts in the central nervous system [lateral hypothalamus and curved nucleus] to generate the feeling of hunger);^27^ and decreased glucagon-like peptide-1 (GLP-1) concentrations in sodium-sensitive individuals when there is an increase in dietary sodium intake (GLP-1 receptors are present in the curved and paraventricular nucleus of the hypothalamus, thus contributing towards reduction of appetite and food intake).^28^ In animal models, high sodium intake increases endogenous production of fructose, thereby triggering the processes of leptin resistance and hyperphagia, which result in obesity, insulin resistance and hepatic steatosis among mice.^29^

Differently from Antonello et al., who also evaluated a population in southern Brazil,^30^ we observed in our study that there was a significant association between STST and BP levels. Our results are in agreement with other studies conducted among different populations.^31^^,^^32^^,^^33^ However, there are some controversies regarding the relationship between the salt taste threshold and BP status: in some studies, patients with hypertension have shown a higher recognition threshold for salt,^30^^,^^34^ while in others it was concluded that there was no difference in STST between people with and without hypertension.^35^^,^^36^ Studies evaluating potential associations between STST and health outcomes are still scarce and quite heterogeneous regarding the type of design used, the methods used to identify BP levels and sodium intake, the type of population and the number of participants evaluated, which makes it difficult to compare the results.

Among the limitations of this study, we can mention the cross-sectional design, which may have presented the bias of reverse causality; the lack of quantification of urinary sodium and non-performance of 24-hour ambulatory blood pressure monitoring (ABPM); the application of a one-day 24-hour recall, which might not have accurately reflected the dietary habits of the sample (it may underestimate or overestimate food intake, or may characterize flat-slope syndrome); no information about the participants’ levels of physical activity was obtained (this is an important factor associated with BP levels); and most of the participants in the study were Caucasians and showed high education levels.

Conversely, we can highlight that our study represented “real life”, since ABPM and urinary analysis are not always widely available in clinical practice. Furthermore, we tried to perform analyses that were adjusted for a range of confounding factors that have been correlated with BP changes.

## CONCLUSION

An association between STST and BP was observed among the healthy adults participating in this study, regardless of other risk factors for elevation of BP levels, such as age, nutritional status and micronutrient intake. In addition, we identified that the energy and sodium intakes were higher among participants with high STST, and these individuals’ BMI and WC were also higher. This result emphasizes the importance of preventive interventions for lifestyle changes, in order to avoid the development of hypertension and other chronic non-communicable diseases.
